# Carboxylesterases in lipid metabolism: from mouse to human

**DOI:** 10.1007/s13238-017-0437-z

**Published:** 2017-07-04

**Authors:** Jihong Lian, Randal Nelson, Richard Lehner

**Affiliations:** 1 Group on Molecular and Cell Biology of Lipids, University of Alberta, Edmonton, Alberta Canada; 2 Department of Pediatrics, University of Alberta, Edmonton, Alberta Canada; 3 Department of Cell Biology, University of Alberta, Edmonton, Alberta Canada

**Keywords:** carboxylesterase, lipase, lipid, lipoprotein, liver, adipose, intestine

## Abstract

Mammalian carboxylesterases hydrolyze a wide range of xenobiotic and endogenous compounds, including lipid esters. Physiological functions of carboxylesterases in lipid metabolism and energy homeostasis *in vivo* have been demonstrated by genetic manipulations and chemical inhibition in mice, and *in vitro* through (over)expression, knockdown of expression, and chemical inhibition in a variety of cells. Recent research advances have revealed the relevance of carboxylesterases to metabolic diseases such as obesity and fatty liver disease, suggesting these enzymes might be potential targets for treatment of metabolic disorders. In order to translate pre-clinical studies in cellular and mouse models to humans, differences and similarities of carboxylesterases between mice and human need to be elucidated. This review presents and discusses the research progress in structure and function of mouse and human carboxylesterases, and the role of these enzymes in lipid metabolism and metabolic disorders.

## INTRODUCTION

Mammalian carboxylesterases (EC 3.1.1.1) belong to a multigene superfamily encoding enzymes that have broad substrate specificity and catalyze the hydrolysis of ester-, thioester-, and amide-bond containing xenobiotic and endogenous compounds. Carboxylesterases are mainly known as enzymes involved in detoxification and metabolism of (pro)drugs and environmental toxicants (reviewed in Hatfield et al., 2016; Fukami et al., 2015; Laizure et al., 2013; Staudinger et al., 2010; Sanghani et al., 2009; Imai, 2006). However, carboxylesterases have also been demonstrated to hydrolyze endogenous esters and thioesters including lipids and some of these enzymes have been shown to play important physiological functions in lipid metabolism and energy homeostasis. Recent research endeavors have provided more insight into the roles of human carboxylesterases in metabolic diseases.

Genes encoding six human carboxylesterases and twenty mouse carboxylesterases have been classified. However, given the interspecies diversity of carboxylesterases both in the number and primary amino acid sequences there is a need to define functional mouse and human orthologs.

This review will discuss the current knowledge of this class of enzymes in mice and humans with emphasis on physiological functions of carboxylesterases in lipid metabolism and human diseases.

## GENE AND PROTEIN NOMENCLATURE OF MAMMALIAN CARBOXYLESTERASES

Mammalian carboxylesterases are a family of proteins encoded by multiple genes. The six human carboxylesterase genes, including one pseudogene, are all localized on chromosome 16. A large number of rodent carboxylesterase genes were generated from tandem gene duplication. Twenty mouse carboxylesterase genes including one pseudogene have been annotated, all located on chromosome 8 (Jones et al., 2013; Williams et al., 2010; Holmes et al., 2010a; Kroetz et al., 1993). Mammalian carboxylesterase genes usually contain 12–14 exons and encode protein products of approximately 60 kDa (Williams et al., 2010; Holmes et al., 2010a).

Early nomenclature of carboxylesterases was based on enzyme characteristics such as substrate specificity or pI value, order of identification, or tentatively named when isolated or sequenced (Sanghani et al., 2009; Furihata et al., 2004; Ellinghaus et al., 1998; Dolinsky et al., 2001; Robbi et al., 1990; Furihata et al., 2003; Strausberg et al., 2002; Ovnic et al., 1991). However, there has been significant confusion in the nomenclature of these genes/enzymes resulting in incorrect ortholog assignments. This is because: (1) Different carboxylesterases show substrate or pI value overlap; (2) Various labs isolated the same carboxylesterase independently and assigned it a different name based on enzymatic activity; (3) There is a significantly larger number of carboxylesterase genes in rodents compared to humans, and this makes mouse/human ortholog assignment challenging. Because mouse models are widely used for functional studies, the confusion of nomenclature and incorrect ortholog assignment has led to incorrect conclusions and misinterpretation in several studies, not only involving mouse-to-human ortholog assignments but also in mouse-to-mouse carboxylesterase identification.

Effort was made to standardize the nomenclature of mammalian carboxylesterases (Holmes et al., 2010a). In this system, mammalian carboxylesterases are grouped into five families based on homology and gene structure/chromosome localization. The guidelines of human, mouse, and rat gene nomenclature committees were followed and the capitalized “CES” root is used for human carboxylesterases, whereas “Ces” is used for mouse and rat carboxylesterases, followed by the family number. Italic *CES*/*Ces* nomenclature is used for genes, while non-italic CES/Ces nomenclature is used for proteins. In the case of multiple genes in a family, a letter is added following the family number. Six human *CES* genes, described in this system as *CES1* (Furihata et al., 2004; Alam et al., 2002a; Riddles et al., 1991), *CES2* (Furihata et al., 2003; Pindel et al., 1997; Schwer et al., 1997), *CES3* (Mori et al., 1999; Sanghani et al., 2004), *CES4A* (Holmes et al., 2009a), *CES5A* (Miyazaki et al., 2006) and a *CES1*-like pseudogene *CES1P1* (Yan et al., 1999) have been assigned so far. Eight genes belonging to the mouse *Ces1* family are localized in tandem cluster on mouse chromosome 8, the names of these genes are assigned in the same order as their locations on the chromosome from *Ces1a* to *Ces1h*. Eight genes of the mouse *Ces2* family are localized on another gene cluster, and similar to the *Ces1* family, they are named according to their order position in the cluster (*Ces2a* to *Ces2h*). There are two *Ces3* genes (*Ces3a* and *Ces3b*), one *Ces4a* gene and one *Ces5a* gene.

An example of how carboxylesterase nomenclature can be confused in literature is as follows. Some studies used the capitalized CES designation for mouse genes/proteins (Xu et al., 2014a, b, 2016). In fact, the confusion becomes even deeper because the old gene nomenclature for *Ces1g* is *Ces1* and when CES1 (gene and protein) was used instead of Ces1 or Ces1g (gene and protein) readers would automatically assume that mouse Ces1g is an ortholog of human CES1. However, the functional mouse ortholog of human CES1 has been demonstrated to be Ces1d (Gilham et al., 2005; Alam et al., 2006; Wei et al., 2010), not Ces1g (Quiroga et al., 2012a). The functional human ortholog for Ces1g [previously Ces1 and also known as Es-x (Ellinghaus et al., 1998)] has not yet been defined. Similarly, a recent report assigned Ces2c, previously annotated as Ces2, as the ortholog of human CES2 (Li et al., 2016). However there are six members of the mouse *Ces2* gene family and it is not even given that the functional mouse ortholog of human CES2 must come from the *Ces2* gene family. Therefore, the functional mouse ortholog of human CES2 remains to be defined. Incorrect ortholog assignments have complicated the understanding of the published literature. The standardized nomenclature method (Holmes et al., 2010a) allocates a unique name and facilitates systematic identification for each of the genes within or across species. In this review the accepted nomenclature system (Holmes et al., 2010a) will be used. Table 1 summarizes the names and according aliases originated from previous studies for mouse carboxylesterases.

**Table 1:** Aliases of mouse carboxylesterases

**Mouse Ces gene/protein**	**Aliases** **gene/protein**
Ces1a	EG244595
Ces1b	Gm5158
Ces1c	Es1 (Genetta et al., 1988)
Ces1d	Ces3, CesMH1, triacylglycerol hydrolase (TGH) (Dolinsky et al., 2001), cholesteryl ester hydrolase (CEH) (Ghosh et al., 1995), Es10/pI6.1 esterase (Robbi et al., 1990), hydrolase A (Morgan et al., 1994)
Ces1e	Egasyn, Es22 (Ovnic et al., 1991)
Ces1f	CesML1, TGH2 (Okazaki et al., 2006)
Ces1g	Ces1, Es-x (Ellinghaus et al., 1998)
Ces2a	Ces6
Ces2c	Ces2 (Furihata et al., 2003)
Ces2e	Ces5
Ces3b	Es31 (Aida et al., 1993)
Ces4a	Ces8
Ces5a	Ces7, Cauxin (Li et al., 2011)

## PROTEIN STRUCTURE AND FUNCTIONAL DOMAINS OF CARBOXYLESTERASES

**Figure 1:**
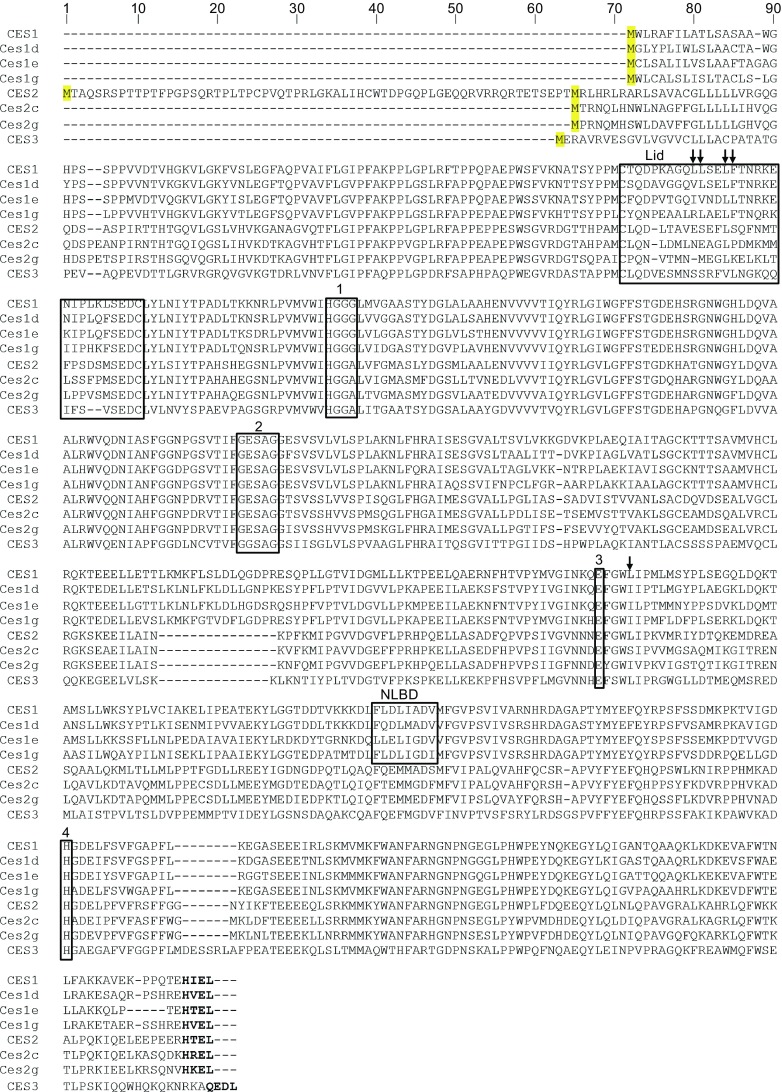
**Amino acid sequence alignments of human and murine carboxylesterases reported to hydrolyze lipids.** Boxed residues indicate conserved functional residues and domains: 1, oxyanion hole-forming domain; 2, GXSXG catalytic serine motif; 3, catalytic glutamic acid; 4, catalytic histidine; NLBD, putative neutral lipid binding domain. The HXEL ER retrieval sequence is indicated with bold letters. Residues that comprise the rigid pocket on CES1 are indicated with arrows. GenBank accession numbers: CES1, NP_001257; CES2, NP_003860; CES3, NP_079198; Ces1d, NP_444430; Ces1e, NP_598421; Ces1g, NP_067431; Ces2c, NP_663578; Ces2g, NP_932116

Carboxylesterases belong to a family of isoenzymes that has been highly conserved during evolution (Williams et al., 2010). Human carboxylesterases share between 39% to 46% amino acid sequence identities (Holmes et al., 2010a). There is also significant interspecies sequence similarity. For example, mouse Ces1d and human CES1 proteins share 78% identity and 88% similarity at the amino acid level (Fig. 1). Amino acid sequence alignments of different carboxylesterase isoenzymes from various species reveal high conservation of key residues and critical domains in protein sequences (Fig. 1). The hydrophobic N-terminal sequence of carboxylesterases shows variability but all contains a functional signal peptide that directs the carboxylesterase protein expression to the lumen of the endoplasmic reticulum (ER) (Potter et al., 1998). Human *CES2* gene has two in-frame ATGs. The use of the first ATG in exon 1 produces a CES2 variant with extra 64 amino acids in the N-terminus. The biological function of the extra 64 amino acids remains to be determined (Sanghani et al., 2009).

**Figure 2:**
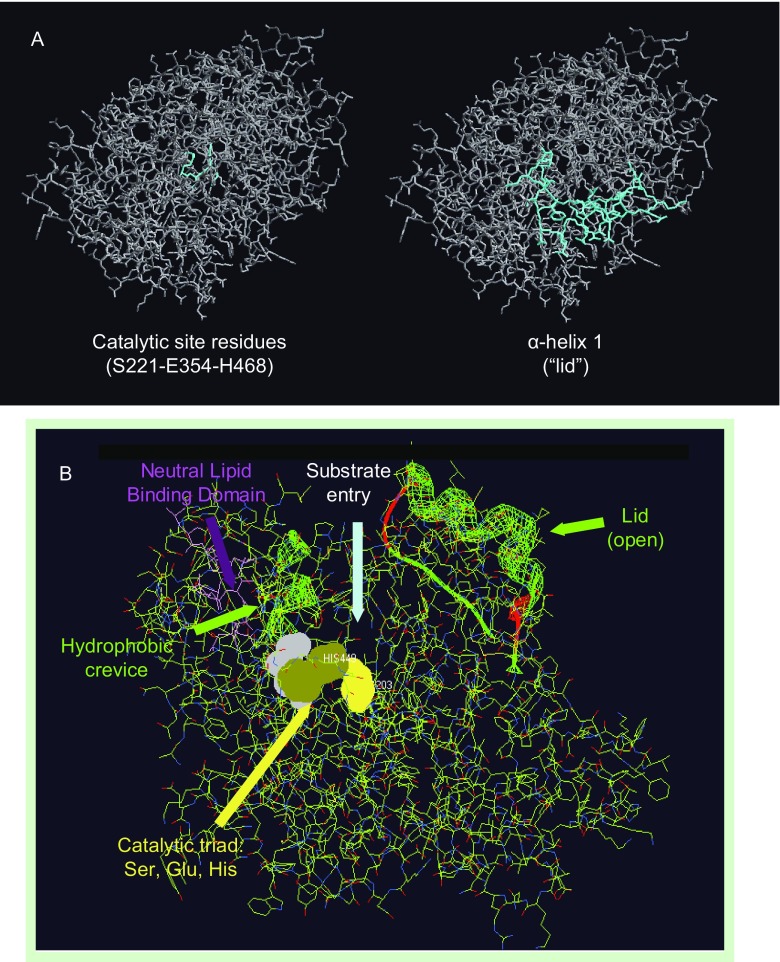
**Three-dimensional structure of human CES1**

Carboxylesterases belong to the α/β-hydrolase fold family of proteins. Murine and human Ces1d/CES1 protein sequences contain 17 α helices and 17 β strands (Dolinsky et al., 2004). The three-dimensional structure of CES1 confirmed the α/β-hydrolase fold comprising a central catalytic domain and adjacent α/β regulatory domains (Bencharit et al., 2002, 2003a; Alam et al., 2002b). X-ray crystal structure of CES1 also confirmed its existence as a monomer, trimer and hexamer, with substrate dependent equilibrium of homooligomer formation (Bencharit et al., 2003b). Predicted secondary structures of other human carboxylesterases, including CES2 and CES3, have suggested similar α/β hydrolase folds (Holmes et al., 2009b, 2010b). The catalytic domain of CES1 encompasses a serine hydrolase catalytic triad that is located at the bottom of a deep active site cleft (Fig. 2). The three residues that form the catalytic triad of carboxylesterases, Ser, Glu, and His, are highly conserved among species and isoenzymes (Fig. 1). The residues in the catalytic triad are Ser^221^, Glu^354^, and His^468^ in human CES1 and Ser^221^, Glu^353^, and His^466^ in mouse Ces1d (Holmes et al., 2010a). Mutation of any of the catalytic triad residues abolishes carboxylesterase activity (Alam et al., 2002b). The active site cleft comprises a large flexible pocket on one side of the catalytic serine and a small rigid pocket on the opposite side (Bencharit et al., 2003b). The large flexible pocket may confer the ability of carboxylesterases to hydrolyze many structurally distinct compounds, whereas the small rigid pocket facilitates selectivity (Bencharit et al., 2003b; Hosokawa 2008). The rigid pocket is lined by hydrophobic residues comprising α-helix 1, which was suggested to act as a “lid” (Fig. 2) (Dolinsky et al., 2004). The location of α-helix 1 is highly conserved among carboxylesterases from various species (Dolinsky et al., 2004) (Fig. 1). However, the amino acid sequences within α-helix 1 diverge among different carboxylesterase isoenzymes, which suggest variability in substrate selectivity of the isoenzymes, and therefore different metabolic function. For example, mouse Ces1d and Ces1g share 76% amino acid sequence identity, however, the sequences of the α-helix 1 domains are distinct (Fig. 1), and these two isoenzymes exhibit very different biological functions (discussed below). Lid domains have been demonstrated to play a vital role in the interfacial activation and in substrate selectivity of lipolytic enzymes, including pancreatic lipase, lipoprotein lipase, and fungal lipases (Carriere et al., 1998; Griffon et al., 2006; Dugi et al., 1995; Brocca et al., 2003). The oxyanion hole formed by Gly^142^ and Gly^143^ in the HGGG motif (motif 1 in Fig. 1) is adjacent to the conserved rigid pocket (Dolinsky et al., 2004; Bencharit et al., 2003b).

Cysteine residues in carboxylesterases are involved in specific disulfide bond formation. Four cysteine residues are present in mouse Ces1d, and five in human CES1. Two of the Cys residues form a bridge that defines α-helix 1 (Fig. 1).

A putative neutral lipid binding domain (NLBD) has been suggested to play a role in the affinity of enzymes containing this motif for neutral lipids (Alam et al., 2006; Dolinsky et al., 2004). There is significant conservation in the NLBD sequence FLXLXXXn (X, any residue; *n* = nonpolar amino acid residue) between human CES1 and mouse Ces1d, Ces1e and Ces1g, but differences, especially the absence of the second Leu residue, are noted in human CES2 and mouse Ces2 family, as well as in human CES3 (Fig. 1).

## INTRACELLULAR LOCALIZATION OF CARBOXYLESTERASES

Carboxylesterases have been described to be present in several subcellular organelles. The majority of carboxylesterases are intracellular proteins found predominantly in the microsomal fraction encompassing the endoplasmic reticulum (ER), and some carboxylesterases are secreted from cells (Furihata et al., 2004; Maki et al., 1991; Hosokawa et al., 1995, 1990). Microsomal carboxylesterases can be released from their membrane-associated state by treatment with carbonate at alkaline pH, which together with the presence of cleavable signal peptide sequence indicates that these enzymes are not transmembrane proteins but soluble proteins that reside in the lumen of the ER. Soluble proteins that reside in the ER lumen of mammalian cells are prevented from secretion by retrieval from the secretory pathway back to the ER by KDEL receptor mediated recognition of a C-terminal KDEL sequence (Pelham 1991; Townsley et al., 1993; Munro and Pelham 1987). Microsomal carboxylesterases from human, mouse, rat, and rabbit carry the HXEL variations of the KDEL consensus ER retrieval sequence at their extreme C-terminal and the HXEL motifs have been shown to be necessary and sufficient for ER retention (Robbi and Beaufay, 1991). For example, mouse Ces1d and human CES1 contain functional HVEL and HIEL retrieval sequences, respectively. On the other hand, human CES3 C-terminal sequence of QEDL does not conform to the standard KDEL or HXEL (Fig. 1), which may affect the localization of this carboxylesterase. CES4 and CES5 that apparently lack the canonical ER retrieval signal are likely to be secreted proteins (Holmes et al., 2009a; Miyazaki et al., 2006). Immunogold electron microscopy, and immunofluorescence imaging confirmed the localization of CES1 in the ER lumen of hepatocytes (Gilham et al., 2005). The formation of disulfide bond and *N-*linked glycosylation are processes that occur in the ER lumen (Bulleid, 2012; Breitling and Aebi 2013). The presence of disulfide bridges and glycosylated residues (Alam et al., 2002b) in Ces1d/CES1 is consistent with their ER-localization. It has been reported that CES1 was associated with cytosolic fraction and cytosolic lipid droplets (CLDs) in macrophages (Zhao et al., 2005). These results were obtained following cell homogenization and subcellular fractionation and therefore there is some possibility that the ER integrity has been disrupted during the homogenization process resulting in leakage of CES1 from the ER. On the other hand, the continuum formed between CLDs and the ER might enable ER lumen localized proteins to interact with CLDs (Wilfling et al., 2014; Mishra et al., 2016). The presence of ER resident proteins BiP (Liu et al., 2004) and calnexin (Brasaemle et al., 2004) on CLDs has been documented, thus it is plausible that lumenal carboxylesterases could gain access to CLDs. However, calnexin is a transmembrane and not a hairpin membrane protein (Ho et al., 1999) and as such it would not be expected to be able to intercalate into the phospholipid monolayer of CLDs. The presence of transmembrane ER proteins such as calnexin in the CLD fraction suggests that during the process of homogenization followed by subcellular fractionation, co-isolation of the ER bridged to CLDs could occur.

Carboxylesterase activity was also identified in rodent plasma (Bahar et al., 2012). In rat and mouse, Ces1c proteins that lack the C-terminal HXEL ER retrieval sequence were shown to be secreted from the liver after their synthesis (Yan et al., 1995; Genetta et al., 1988). Therefore, in general, mammalian carboxylesterase synthesis is directed to the lumen of the ER where their signal sequences are cleaved, and the proteins are disulfide bonded and glycosylated. Carboxylesterases that contain the canonical ER retrieval sequence become lumenal ER residents (and may associate with cytosolic or lumenal LDs), while carboxylesterases without the ER retrieval signal are secreted out of the cell.

## TISSUE DISTRIBUTION AND SUBSTRATES OF CARBOXYLESTERASES

Carboxylesterases are expressed in many tissues, however, specific tissues express specific isoforms. In humans, the two predominant carboxylesterases CES1 and CES2 are abundantly expressed in liver and intestine, respectively (Jones et al., 2013; Williams et al., 2010), the two organs that are responsible for first pass clearance of xenobiotics, but also the organs that are most active in lipoprotein secretion. CES1 is also expressed in the adipose tissue, kidney, heart, and macrophages (Sanghani et al., 2009; Hosokawa et al., 1995; Ghosh 2000; Satoh et al., 2002). CES2 exhibits more specific tissue expression and is mainly expressed in the intestine with lower expression in the liver. Similar to CES2, *CES3* mRNA is specifically expressed in the liver and intestine, but in relatively lower abundance compared to *CES1* and *CES2* (Sanghani et al., 2004). Mouse Ces1d and its human ortholog CES1 have similar tissue/cell protein expression profiles, with the exception of macrophages where Ces1d shows minimal or no expression, while CES1 protein is significantly expressed (Jones et al., 2013; Okazaki et al., 2008). Each mouse carboxylesterase within the same subfamily exhibits relatively unique expression pattern compared with other members. For example, Ces1g has a more specific tissue distribution than Ces1d, and is significantly expressed only in the liver and intestine (Quiroga et al., 2012a). Mouse *Ces2* gene family is more specifically and abundantly expressed in the intestine compared to other organs (Jones et al., 2013; Fu et al., 2016).

Carboxylesterases have a broad substrate specificity including compounds with ester, amide, or thioester bonds. CES1 and CES2 have been extensively investigated for their roles in (pro)drug metabolism. Although they share 47% amino acid identity, CES1 and CES2 exhibit distinct substrate specificities. CES1 was demonstrated to mainly hydrolyze substrates with small alcohol groups and large acyl groups, CES2 was shown to hydrolyze substrates with a large alcohol group and small acyl group (Hosokawa, 2008). As an example, CES1-specific substrates include narcotics, clopidogrel, meperidine, delapril, and methylphenidate; CES2 shows more activity toward aspirin, the anticancer prodrug irinotecan (CPT-11), and flutamide (Sanghani et al., 2009; Imai, 2006; Bencharit et al., 2002; Imai et al., 2006). The substrate specificity of the other human carboxylesterases has not been studied extensively. CES3 also has been reported to hydrolyze CPT-11 but shows much lower activity when compared with CES2 (Sanghani et al., 2004).

In addition to xenobiotics, carboxylesterases also hydrolyze endogenous lipids. The role of carboxylesterases as lipid hydrolases (lipases) functioning in energy homeostasis and human metabolic diseases has attracted substantial research interest. Both CES1 and CES2 were demonstrated to possess triacylglycerol (TG) hydrolase activity (Alam et al., 2002a; Ruby et al., 2017). Diacylglycerol (DG) hydrolase activity of CES2 has also been reported (Ruby et al., 2017). Besides the mouse ortholog of human CES1, Ces1d, other mouse carboxylesterases including Ces1f (previously TGH-2) (Okazaki et al., 2006), Ces1g (Quiroga et al., 2012a; Ko et al., 2009) and Ces2c (previously Ces2) (Li et al., 2016) also have been demonstrated to harbor TG hydrolase activities. Cholesteryl ester (CE) hydrolase activity of CES1 in human macrophages has been reported (Ghosh, 2000; Crow et al., 2010), but CE hydrolase activity of CES1 could not be demonstrated by other research groups (Igarashi et al., 2010; Buchebner et al., 2010). CES1 also exhibits hydrolase activity toward endocannabinoid 2-arachidonoylglycerol (2-AG) and its cyclooxygenase (COX)-derived prostaglandin glyceryl esters in human THP-1 monocytes/macrophages (Xie et al., 2010; Wang et al., 2013). Mouse Ces2g is expressed in the spleen and exhibits 2-AG hydrolase activity as well. In response to inflammatory stimuli, Ces2g expression in the spleen is decreased with an accompanying reduction of 2-AG hydrolase activity (Szafran et al., 2015).

Several carboxylesterases harbor retinyl ester (RE) hydrolase activity and may be thus involved in hepatic RE metabolism. Rat Ces1c, Ces1d, Ces1e, and Ces1f have all shown RE hydrolase activity in *in vitro* assays (Mentlein and Heymann, 1987; Linke et al., 2005; Sun et al., 1997; Sanghani et al., 2002). Chylomicron remnant-associated RE have been proposed to be taken up by hepatocytes through receptor-mediated endocytosis followed by transfer of RE to the ER rather than to lysosomes. In this process, RE undergoes hydrolysis after uptake into the hepatocyte (Harrison et al., 1995). Rat liver expresses Ces1c (Yan et al., 1995) and this carboxylesterase was identified as a neutral, bile salt-independent RE hydrolase in the liver microsomal fraction (Sun et al., 1997). However, Ces1c lacks the C-terminal ER retention/retrieval sequence and was demonstrated to be one of the secreted carboxylesterases (Yan et al., 1995). These data would suggest that Ces1c could be involved in the RE hydrolysis in early endosomes and/or function on chylomicron RE at or near the cell surface in the space of Disse (Sun et al., 1997). On the other hand, Linke et al., (2005) reported that rat Ces1d possesses neutral and acid RE hydrolase activity in the liver microsomal fraction, and suggested that Ces1d could play a role in the hydrolysis of endocytosed chylomicron RE in both neutral and acidic membrane compartments of hepatocytes. Mouse Ces1e is highly expressed in the liver and exhibits robust RE hydrolase activity (Schreiber et al., 2009). Overexpression of Ces1e in Cos-7 cells inhibited RE accumulation. Instead of mobilizing RE stores contained in CLDs, Ces1e was shown to affect RE metabolism by counteracting retinol esterification enzymes (Schreiber et al., 2009). Notably, in this study, overexpression of mouse Ces1d in Cos-7 cells did not correlate with significant increase in RE hydrolase activity, while expression of Ces1c and Ces1f coincided with increased RE hydrolase activity (Schreiber et al., 2009). These data therefore appear to challenge the role of Ces1d in RE metabolism. While several carboxylesterases appear to possess RE hydrolase activity, more research is required to address the physiological significance of these carboxylesterases in RE metabolism.

## SINGLE NUCLEOTIDE POLYMORPHISMS (SNPS) OF HUMAN CARBOXYLESTERASES

Single nucleotide polymorphisms (SNPs) have been identified in human carboxylesterases (Kim et al., 2003; Saito et al., 2003; Wu et al., 2004; Zhu et al., 2008; Yamada et al., 2010). Some of the SNPs are localized in the promoter or coding regions that affect protein expression or enzyme activity. Particularly, a coding SNP (GGG to GAG) in *CES1* exon 4 results in Gly^143^Glu substitution. Gly143 resides in the oxyanion hole-forming domain (HGGG^143^) that plays an important role in CES1 catalytic activity. Ectopic expression of CES1 carrying this mutation confirmed significant reduction of its esterase activity *in vitro* (Zhu et al., 2008). The minor allele frequency of Gly^143^Glu was determined to be 3.7%, 4.3%, 2.0%, and 0% in Caucasian, Black, Hispanic, and Asian populations, respectively. A deletion in exon 6 at codon 260 results in a frameshift mutation and complete loss of hydrolytic activity. The Asp260fs appears to be a very rare mutation (Zhu et al., 2008). A SNP A^(−816)^C localized in the promoter region of *CES1* gene increases transcriptional efficiency (Geshi et al., 2005). Patients carrying the A^(−816)^C SNP showed enhanced anti-hypertension response to the angiotensin-converting enzyme (ACE) inhibitor imidapril, which is converted to its active metabolite, imidaprilat, by CES1 (Geshi et al., 2005). CES1 inactivates the antiplatelet agent clopidogrel through ester hydrolysis. The A^(−816)^C variation attenuates responsiveness to clopidogrel in patients diagnosed with coronary heart disease. The A^(−816)^C polymorphism was not observed to be significantly associated with stent thrombosis occurrence in this study (Xie et al., 2014).

Notably, allele frequencies and estimated haplotype frequencies of SNPs in human carboxylesterases varied significantly in different populations (Kubo et al., 2005; Marsh et al., 2004). The physiological significance of SNPs in human carboxylesterases on lipid metabolism and energy homeostasis has not yet been fully elucidated.

## PHYSIOLOGICAL FUNCTIONS OF CARBOXYLESTERASES IN LIPID METABOLISM AND METABOLIC DISEASES

### Regulation of cytosolic lipid droplets (CLDs) metabolism by carboxylesterases

Lipid droplets (LDs) are dynamic intracellular organelles implicated in many cellular functions, including lipid storage and mobilization, protein storage and degradation, lipid mediated cell signaling and others (Walther and Farese, 2012). Cellular energy is stored in LDs mainly in the form of TG. In mammalian cells, CLDs are comprised of a neutral lipid core containing mainly TG with some CE and RE surrounded by a monolayer of amphipathic lipids (phospholipids and free cholesterol) and LD-associated proteins (Martin and Parton, 2006). Abnormalities in CLD dynamics have been implicated in human diseases such as obesity, cardiovascular disease, type 2 diabetes, and fatty liver diseases. Although white adipose tissue is the most predominant tissue for lipid storage, CLDs are present in nearly all cells and tissues. Liver has the second largest capacity to store lipids in CLDs next to adipose tissue. It is generally believed that CLD biogenesis in eukaryotes initiates from the ER where TG biosynthesis takes place (Walther and Farese, 2009). Ces1d expression has been shown to associate with changes in CLD dynamics. In mouse hepatocytes, Ces1d deficiency does not affect the formation of nascent LDs on the ER, but results in decreased size and increased number of CLDs by reducing the rate of lipid transfer to preformed CLDs (Wang et al., 2010; Lian et al., 2012a). Correspondingly, ectopic expression of the human Ces1d ortholog CES1 results in the formation of large CLDs (Blais et al., 2010).

### Role of carboylesterases in lumenal lipid droplet (LLD) metabolism and lipoprotein assembly and secretion

**Figure 3:**
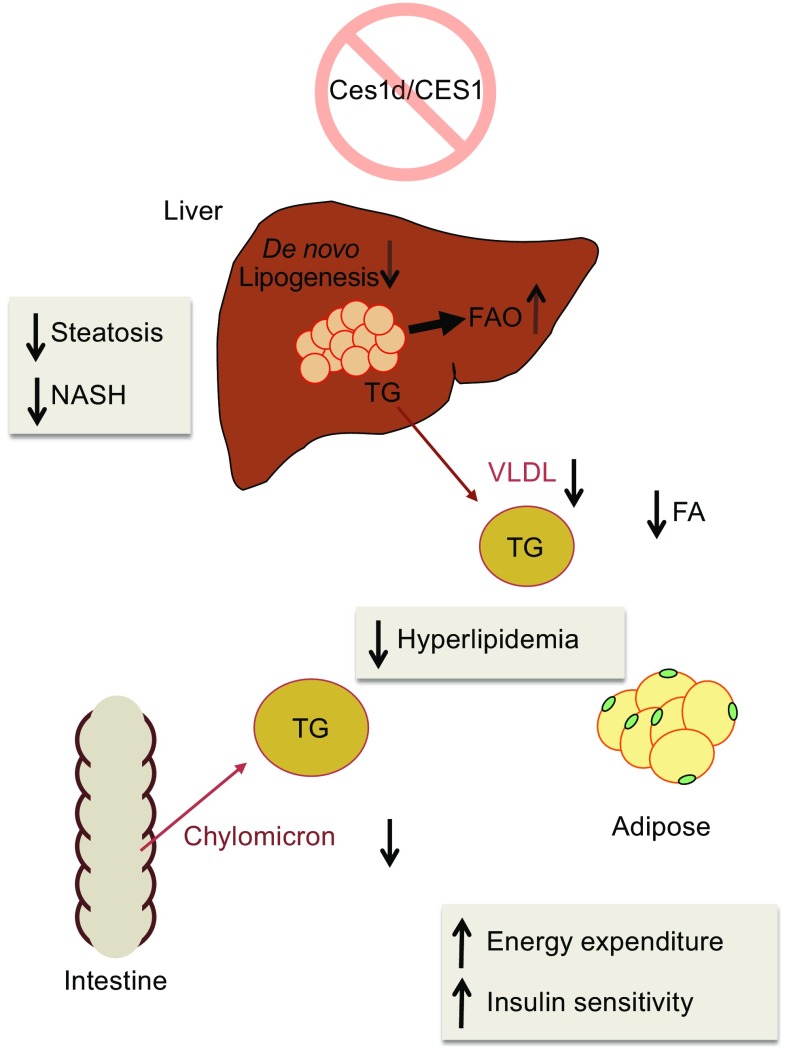
**Effects of Ces1d deficiency on lipid and energy homeostasis**

In addition to CLDs, hepatocytes synthesize ER lumenal apoB-free LDs (LLDs), and apoB-containing very-low density lipoprotein (VLDL) particles (Lehner et al., 2012; Alexander et al., 1976). The proposed function of LLDs is to provide a pool of TG for VLDL assembly (Lehner et al., 2012; Gibbons et al., 2004). Ces1d was shown to associate with LLDs within the ER lumen (Wang et al., 2007), suggesting a role of Ces1d in the mobilization of lumenal TG for the VLDL assembly process. It is well accepted that oversecretion of apoB—containing lipoproteins, chylomicrons from the intestine and VLDL from the liver, contributes to hyperlipidemia and cardiovascular complications. The current model of chylomicron and VLDL assembly proposes a two-step process (Shelness and Sellers, 2001; Innerarity et al., 1996; Olofsson et al., 2000; Wiggins and Gibbons, 1992). In the first step, newly synthesized apoB is lipidated during its translocation across the ER into the lumen yielding a primordial apoB particle. In the second step, bulk transfer of core lipids from LLDs to the primordial apoB particle is believed to take place posttranslationally. It has been hypothesized that the ER lumen localized LLD-associated Ces1d functions to mobilize lipids to provide substrates for VLDL assembly through a process of “hydrolysis/re-esterification cycle” (Lehner et al., 2012; Wang et al., 2007). It has been shown that overexpression of Ces1d or its human ortholog CES1 increases hepatic VLDL secretion whereas inhibition of Ces1d decreases hepatic VLDL secretion both *in vitro* (Gilham et al., 2003; Lehner and Vance, 1999) and *in vivo* (Wei et al., 2010, 2007a; Lian et al., 2012a, 2016). In addition, Ces1d deficient mice exhibit decreased chylomicron secretion (Lian et al., 2012b). Consequently, Ces1d knockout mice present with decreased plasma lipid levels (Wei et al., 2010; Lian et al., 2012a, b) (Fig. 3).

**Figure 4:**
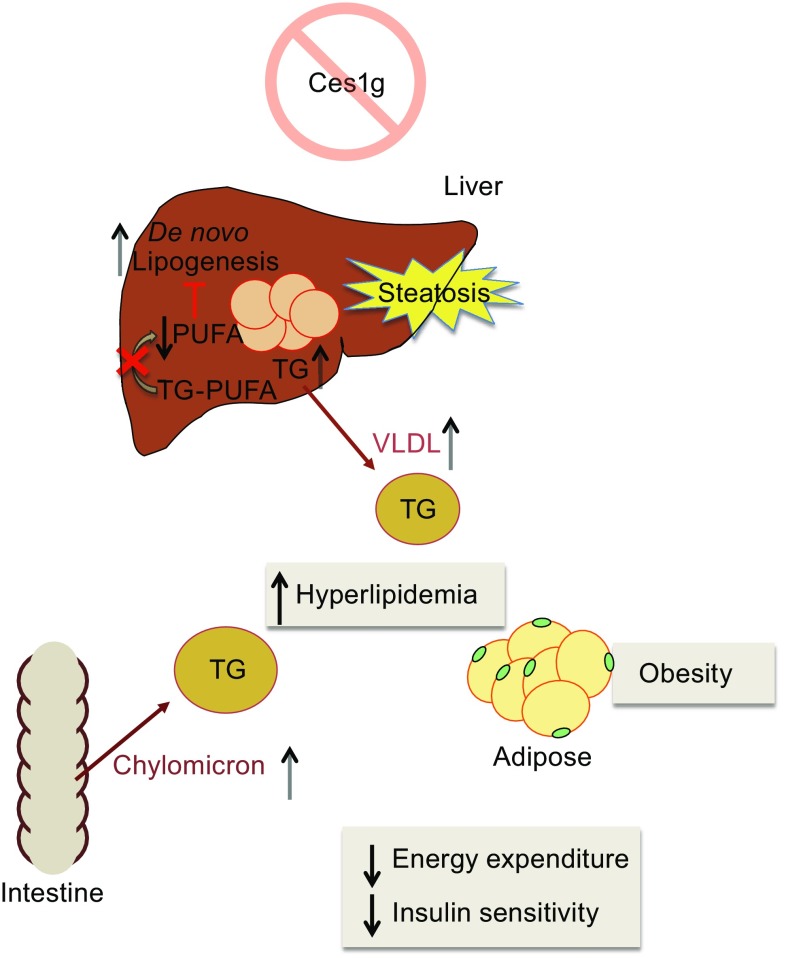
**Effects of Ces1g deficiency on lipid and energy homeostasis**

Another carboxylesterase in the mouse Ces1 family, Ces1g, affects lipoprotein metabolism in a very different fashion from Ces1d. Ablation of Ces1g expression *in vivo* results in both postabsorptive (fasting) and postprandial hyperlipidemia and augmented circulating apoB concentrations due to increased secretion of VLDL (Quiroga et al., 2012a) and chylomicrons (Quiroga et al., 2012b) (Fig. 4). Furthermore, analysis of apolipoprotein profiles from the blood of Ces1g deficient mice showed protein composition changes, including increased apoE and apoCIII (an endogenous inhibitor of lipoprotein lipase (LpL)) and decreased apoCII levels (an endogenous activator of LpL), which can cause blunted blood apoB-containing lipoprotein clearance and contribute to the observed hyperlipidemia. Restoration of hepatic Ces1g expression in the Ces1g knockout mice reversed hyperlipidemia and fatty liver (Bahitham et al., 2016).

### Carboxylesterases and metabolic diseases

Metabolic disturbances that clinically manifest as elevated blood pressure, dyslipidemia, hyperglycemia, hyperinsulinemia, and obesity increase the risks of numerous human diseases including cardiovascular disease, fatty liver disease, type 2 diabetes mellitus, and cancer (Alberti et al., 2009; Basen-Engquist and Chang, 2011; Faulds and Dahlman-Wright, 2012). The prevalence of metabolic diseases has been increasing and will continue to rise markedly during the coming decades. Dysregulation of lipid metabolism in the body, including synthesis, storage, and catabolism of intracellular lipids, and lipoprotein secretion and clearance in the circulation, contributes to the development of the metabolic diseases. Several isoenzymes in carboxylesterase family have shown to harbor lipase activity and have been demonstrated to be involved in lipid metabolism. The roles of carboxylesterases in the development of metabolic disease have been investigated by numerous studies.

#### Functions of carboxylesterases in adipose tissue and obesity

Insulin resistance is associated with increased adipose tissue-derived circulating fatty acid and ectopic lipid accumulation (Karpe et al., 2011; McQuaid et al., 2011), thus approaches that block excessive fatty acid release from adipose tissue and restore normal tissue lipid partitioning often improve insulin sensitivity (Fulcher et al., 1992). Ces1d is expressed in 3T3-L1 adipocytes (Wei et al., 2005; Dolinsky et al., 2003; Wei et al., 2007b) and adipose tissue (Soni et al., 2004; Birner-Gruenberger et al., 2005). Because of its intracellular localization, Ces1d is expected to play a different role from other cytosolic lipases in adipose tissue such as adipose triglyceride lipase (ATGL) and hormone-sensitive lipase (HSL) (Schweiger et al., 2006). Expression of Ces1d was induced during 3T3-L1 adipocyte differentiation (Dolinsky et al., 2003). Attenuation of Ces1d activity resulted in decreased basal but not isoproterenol-stimulated efflux of fatty acids from 3T3-L1 adipocytes (Wei et al., 2007b). In a phenotypic and activity-based protein profiling (ABPP) screening for small molecules that show activity in a cell-based assay measuring differentiation and lipid accumulation in adipocytes, a subset of bioactive inhibitory compounds that target Ces1d was identified (Dominguez et al., 2014). Administration of Ces1d inhibitors to high-fat diet fed mice or *db*/*db* mice protected from weight gain, reduced plasma lipids, ameliorated liver steatosis, and improved glucose tolerance (Dominguez et al., 2014). Importantly, this study also showed that in the adipose tissue of obese and type 2 diabetic patients, the activity of CES1 is elevated, which is consistent with other studies showing that *CES1* expression is higher in adipose tissue from obese patients compared to lean subjects (Steinberg et al., 2007; Jernas et al., 2009). It has been reported that *CES1* mRNA abundance was positively correlated with clinical parameters of adiposity, which also suggests a role of CES1 in the development of obesity-associated phenotypes (Nagashima et al., 2011; Marrades et al., 2010). These studies confirmed results from the Ces1d knockout mice that presented with decreased blood fatty acids, increased energy expenditure, and improved insulin sensitivity (Wei et al., 2010).

Another carboxylesterase that was observed to show lipase activity in adipose tissue is Ces1f (Okazaki et al., 2006). Ces1f has similar tissue distribution and subcellular localization as Ces1d. Attenuation of Ces1f expression in 3T3-L1 adipocytes resulted in small but significant decrease in glycerol release from isoproterenol-stimulated cells (Okazaki et al., 2006). The physiological function of Ces1f in other tissues where it is expressed has not been reported.

#### Role of carboxylesterases in atherogenesis

Elevated circulating levels of apoB-containing lipoproteins LDL, VLDL, chylomicrons, and chylomicron remnants are recognized independent risk factors for the development of atherosclerosis (Young and Parthasarathy, 1994). Hepatic secretion of VLDL is one of the major determining factors of plasma apoB concentration. Ces1d has been demonstrated to participate in the provision of substrates for VLDL assembly and inactivation of Ces1d decreases VLDL secretion and blood lipid levels *in vivo* (Wei et al., 2010). In an atherosclerotic mouse model, high-fat, high-cholesterol diet fed *Ldlr* ^−/−^ mice, Ces1d deficiency reduced circulating apoB-containing lipoproteins, ameliorated hyperlipidemia and atherosclerotic lesions in the aorta. Notably, the improved insulin sensitivity observed in Ces1d deficient mice could also contribute to reduced atherosclerosis (Lian et al., 2012b). In humans, *CES1* mRNA expression was positively correlated with blood TG concentrations and total/HDL cholesterol (Marrades et al., 2010).

Several studies (Ghosh et al., 2003; Zhao et al., 2007; Bie et al., 2013) have suggested that CES1 in human macrophages hydrolyzes CE to facilitate free cholesterol efflux, and CES1/Ces1d in the liver hydrolyzes high-density lipoprotein (HDL)-CE and promotes cholesterol excretion and reverse cholesterol transport. In these studies, macrophage-specific overexpression of human CES1 reduced atherosclerosis in *Ldlr* ^−/−^ mice (Zhao et al., 2007), and liver-specific deficiency of Ces1d increased atherosclerosis in *Ldlr* ^−/−^ mice (Bie et al., 2013). However, the CE hydrolytic activity of CES1 has been challenged (Igarashi et al., 2010) because while overexpression of CES1 in cholesterol-loaded human THP-1 macrophages increased esterase activity it did not augment CE hydrolase activity and attenuation of *CES1* expression in THP-1 cells by RNAi failed to decrease CE hydrolase activity.

#### Role of carboxylesterases in cancer progression

Dyslipidemia and obesity are associated with increased human cancer mortality and poor cancer outcomes (Basen-Engquist and Chang, 2011; Calle et al., 2003; Alikhani et al., 2013). Rapidly proliferating tumor cells generally require high amounts of fatty acids and cholesterol (Beloribi-Djefaflia et al., 2016), and tumor grafts in mouse models were observed to induce hyperlipidemia by increasing VLDL production and decreasing chylomicron/VLDL turnover to benefit tumor growth (Huang et al., 2016; Brenneman et al., 1975). Tumor-induced hyperlipidemia was attenuated in Ces1d knockout mice resulting in suppression of tumor growth (Huang et al., 2016), suggesting that Ces1d-mediated increase in plasma lipids could promote tumor growth. In another study, decreased hepatic Ces1d was reported in chemically induced hepatocarcinoma in rats, where fish oil supplementation restored Ces1d expression and prevented cancer development (Quiroga et al., 2016). In this study, the observed Ces1d reduction was disassociated from reduced VLDL secretion, which was at least partially due to the elevated MTP abundance in the liver of this cancer model. Since fish oil supplementation has been demonstrated to suppress tumor growth by various mechanisms (Grimble et al., 2002; Larsson et al., 2004), forced expression to restore Ces1d level in the liver of hepatocellular carcinoma models could provide more direct information on whether Ces1d affects liver cancer development. The precise mechanism on how hepatocyte malignancy regulates *Ces1d* expression also requires more investigation.

#### Role of carboxylesterases in fatty liver disease

Nonalcoholic fatty liver disease (NAFLD) is the leading cause of chronic liver injury. NAFLD is commonly associated with insulin resistance, type 2 diabetes, and cardiovascular disease. Clinical phenotypes of NAFLD extend from simple steatosis, which is characterized by excess deposition of TG in the liver, to nonalcoholic steatohepatitis (NASH), which is distinguished from simple steatosis by the presence of hepatocyte injury (ballooning and cell death), inflammation and/or fibrosis. NASH can further progress to liver cirrhosis and hepatocellular carcinoma (Cohen et al., 2011; Tiniakos et al., 2010). Inactivation of Ces1d protected mice from high-fat diet induced steatosis. Ablation of Ces1d expression in two independent NASH mouse models, phosphatidylethanolamine *N*-methyltransferase knockout mice fed high-fat diet, and *Ldlr* ^−/−^ mice fed high-fat, high-cholesterol Western-type diet, reduced liver inflammation, oxidative stress and fibrosis (Lian et al., 2012a, 2016). The protective effect of Ces1d deficiency against liver steatosis is attributed to decreased hepatic *de novo* lipogenesis, increased fatty acid oxidation, and improved insulin sensitivity (Lian et al., 2012a, b, 2016).

While inhibition/ablation of Ces1d activity has a positive effect on lipid and energy metabolism, Ces1g knockout mice present with increased weight gain, hyperinsulinemia, insulin resistance, and decreased energy expenditure (Quiroga et al., 2012a). Ces1g is not expressed in adipose tissue, so the metabolic syndrome phenotype resulting from inactivation of Ces1g is most likely caused by elevated circulating VLDL and chylomicrons (Quiroga et al., 2012a). This is supported by data showing that overexpression of Ces1g in the liver of *ob*/*ob* mice lowered blood glucose concentration and improved insulin sensitivity (Xu et al., 2014a). Ectopic expression of Ces1g in McArdle-RH7777 cells attenuated cellular TG accumulation and increased fatty acid oxidation (Ko et al., 2009), while Ces1g knockout mice developed liver steatosis even on chow diet (Quiroga et al., 2012a). The increased lipid accumulation in Ces1g deficient mice was attributed to activation of hepatic SREBP1c processing leading to increased lipogenesis. Ces1g exhibits specificity for polyunsaturated fatty acids (PUFAs)-containing TG. PUFAs suppress the activity of SREBP1c promoter (Deng et al., 2002), enhance the degradation of *Srebf1* mRNA (Xu et al., 2001) as well as attenuate Insig1 degradation (Lee et al., 2008) and thus negatively regulate *de novo* lipogenesis. Ces1g deficiency decreased PUFA release from TG, which consequently caused sustained SREBP1c activation and increased *de novo* lipogenesis in the liver (Quiroga et al., 2012a) (Fig. 4). Conversely, overexpression of Ces1g in the liver of *ob*/*ob* mice lowered hepatic TG (Xu et al., 2014a). Another study reported that alcohol reduced liver expression of Ces1g and that inactivation of Ces1g aggravated alcohol and methionine and choline deficient (MCD) diet induced hepatitis (Xu et al., 2016).

The role of Ces2c in NAFLD has also been studied. Liver expression of Ces2c is decreased in *db*/*db* mice and high-fat diet fed mice (Li et al., 2016). Restoration of liver Ces2c expression in these models ameliorated obesity and liver steatosis, and improved glucose tolerance and insulin sensitivity, while inactivation of Ces2c in mice induced liver steatosis and liver damage (Li et al., 2016). This study also suggested that in the liver, fatty acids released from Ces2c mediated TG hydrolysis increased fatty acid oxidation and inhibited SREBP1c to decrease *de novo* lipogenesis. However, the physiological function of fatty acids is related to their molecular species. Fatty acid molecular species released from Ces2c catalyzed lipolysis have not been characterized (Li et al., 2016). Nevertheless, attenuation of Ces2c activity appears to have similar effects on metabolism as attenuation of Ces1g activity. It will be important to delineate the precise contribution of Ces2c and Ces1g to the regulation of lipid metabolism because Ces2c does not appear to compensate for the loss of Ces1g, and vice versa.

Human CES2 displays TG and DG hydrolase activity. Decreased human CES2 activity was found in livers from obese people (Ruby et al., 2017). CES2 activity has a strong inverse correlation with HOMA-IR and liver DG concentration. Overexpression of CES2 in the liver of high-fat diet fed mice reduced adipose tissue deposits, improved glucose tolerance and insulin sensitivity (Ruby et al., 2017). CES2 also appears to be involved in the progression of NAFLD. CES2 protein levels were decreased in the livers of NASH patients (Li et al., 2016). Overexpression of CES2 in C57BL/6 mice reversed high-fat diet-induced steatosis. This CES2-mediated decrease of liver TG accumulation coincided with decreased liver lipogenic gene expression and increased fatty acid oxidation. CES2 overexpression in mice suppressed liver inflammation. Increased ER stress was observed in livers of CES2 overexpressing mice, which was dissociated from the ameliorated fatty liver and inflammation (Ruby et al., 2017). Therefore, expression of CES2 appears to have a similar effect on lipid metabolism as expression of Ces2c or Ces1g. For designing and translating pre-clinical studies from mouse models to human, it will be important to determine which one of the two mouse carboxylesterases (Ces1g or Ces2c) is the mouse ortholog of human CES2. Metabolic phenotypes of various mouse transgenic/knockout models are summarized in the Table 2.

**Table 2:** Summary of metabolic phonotypes of various carboxylesterase genetic mouse models

**Genetic models**	**Metabolic phonotypes**
Ces1d knockout mice	Increased energy expenditure and improved insulin sensitivity (Wei et al., 2010; Lian et al., 2012b) Decreased VLDL secretion and improved hyperlipidemia (Wei et al., 2010; Lian et al., 2012a, b) Attenuated steatosis and NASH (Lian et al., 2016) Reduced atherosclerosis (Lian et al., 2012b) Attenuated tumor-induced hyperlipidemia, inhibited tumor growth (Huang et al., 2016)
CES1 liver-specific transgenic mice	Increased VLDL secretion (Wei et al., 2007a)
CES1 macrophage-specific transgenic mice	Reduced atherosclerosis (Zhao et al., 2007)
Ces1g knockout mice	Obesity, insulin resistance, decreased energy expenditure (Quiroga et al., 2012a) Increased VLDL secretion and hyperlipidemia (Quiroga et al., 2012a) Increased chylomicron secretion (Quiroga et al., 2012b) Increased steatosis (Quiroga et al., 2012a) and alcohol-induced hepatitis (Xu et al., 2016)
Ces2c knockdown mice	Increased steatosis (Li et al., 2016)
CES2 liver-specific overexpression	Improved insulin sensitivity and glucose tolerance, reduced steatosis (Ruby et al., 2017)

### Role of CES1 in hepatitis C virus (HCV) propagation

The life cycle of HCV is closely associated with the metabolism of lipids and lipoproteins (Aizawa et al., 2015). CLDs are involved in the production of infectious HCV particles (Miyanari et al., 2007). HCV maturation occurs in the ER and post-ER compartments and VLDL assembly machinery in the host hepatocytes facilitates HCV particles secretion (Gastaminza et al., 2008; Huang et al., 2007). An ABPP screening revealed CES1 as a differentially active enzyme in Huh7 cells replicating HCV (Blais et al., 2010). HCV infection also correlated with high level of endogenous CES1 in transgenic mice containing human-mouse chimeric livers. Overexpression of CES1 increased apoB secretion and abundance of large LDs in Huh7 cells. The knockdown of CES1 in Huh7 cells results in lower level of HCV replication. This study suggested that HCV modulates CES1 activity to create a favorable environment for its efficient propagation in the host (Blais et al., 2010).

## REGULATION OF CARBOXYLESTERASE EXPRESSION AND ACTIVITY

The precise mechanism by which the expression of carboxylesterases is regulated in the context of energy and metabolic homeostasis is not yet fully understood.

Expression of Ces1d and Ces1g proteins in the liver were reduced in mice with combined CGI-58 and ATGL deficiency, and partially reversed by the treatment of peroxisome proliferator-activated receptor α (PPARα) agonist WY-14643 (Lord et al., 2016). Another study reported that the expression of Ces1d was induced during 3T3-L1 adipocyte differentiation. This expression appears to be regulated by the interaction between CCAAT/enhancer-binding protein α (C/EBPα) and the promoter region of the *Ces1d* gene to enhance its transcription (Wei et al., 2005). The binding region on the promoter (distal promoter region) is specifically important for *Ces1d* gene regulation in adipocytes but not in other cell types (Wei et al., 2005).

Diet supplementation with the bile salt cholic acid or with bile acid-binding resin cholestyramine induced hepatic expression of *Ces1g* mRNA (Ellinghaus et al., 1998). Another study also showed that *Ces1g* mRNA level was induced by cholic acid or an FXR agonist. This study also suggested that *Ces1g* was a direct target of FXR, and might be involved in the regulation of liver lipid homeostasis by FXR (Xu et al., 2014a).

In a study that evaluated the regulation of mouse carboxylesterase genes expression by various nuclear hormone receptors (NHR) (Jones et al., 2013), PPARα agonist increased liver mRNA expression of *Ces1d*, *Ces1e*, *Ces1f*, and *Ces2c*. PPARβ activation increased the expression of *Ces1e* and *Ces2e*. *Ces2c* was the most responsive hepatic carboxylesterase to NHR activation in the test, its expression was significantly increased by RXR, PPARα, LXR, and CAR agonists. Interestingly, in the mucosa of the duodenum, *Ces2c* mRNA expression was unaffected by most of the NHR agonists and was only significantly upregulated by a PXR agonist. The different response of carboxylesterase genes to NHR agonists in various organs suggests tissue-specific regulation.

It has been reported that mRNAs of mouse *Ces1* gene family are substrates of regulated IRE1-dependent decay (RIDD) and are degraded under the condition of IRE1 hyperactivation (So et al., 2012). *Ces1d* appears to also be a direct target of miR155, and liver-specific overexpression of miR155 reduced Ces1d abundance, plasma lipids and attenuated high-fat diet induced hepatic steatosis in mice (Lin et al., 2015).

Very limited knowledge exists about the regulation of carboxylesterase protein expression and activity. Interestingly, unlike the reported induction of mouse *Ces1d* mRNA expression by PPARα agonism, Ces1d protein abundance did not appear to be regulated by PPARα. Ces1d protein abundance did not increase by clofibrate administration to wild-type C57BL/6 mice and did not decrease in PPARα deficient mice (Dolinsky et al., 2003). This suggests additional regulation at the level of protein stability/turnover. While the carboxylesterase protein abundance following forced their expression in cells/mice appeared to directly correlate with their hydrolytic activities toward model substrates (Ko et al., 2009; Wei et al., 2007a), studies in human liver samples indicated that CES1 protein abundance did not correlate well with its ability to hydrolyze the CES1-specific substrate bioresmethrin (Ross et al., 2012). The reason for the differential CES1 activities is not clear but it was proposed that these could be due to specific coding SNPs, alternative splice sites or differences in posttranslational modifications. Alternatively, different human samples may contain variable amounts of endogenous substrates and/or inhibitors that may compete with hydrolysis of exogenously provided substrates. No endogenous protein co-factors (activators/inhibitors) for carboxylesterases have yet been described. ApoE was found to be associated with Ces1d on LLDs in the ER lumen (Wang et al., 2007). However, whether apoE modulates Ces1d function in the ER and regulates mobilization of LLD lipids for VLDL assembly and secretion requires further investigation.

## FUTURE DIRECTIONS

Although the roles of carboxylesterases in lipid metabolism and energy homeostasis have been described in various studies, the mechanisms by which carboxylesterases exert their effects, their precise substrate specificity and the identity of potentially biologically active metabolites that are produced as consequence of carboxylesterase activity remain to be determined. The regulation of carboxylesterase expression and activity is also not yet fully understood.

Several carboxylesterases appear to be potential pharmacological targets for the treatment of metabolic disorders and obesity-related complications. Because opposing metabolic functions have been described for some carboxylesterases, development of carboxylesterase isoenzyme specific inhibitors is required. Screening of specific Ces1d/CES1 inhibitors has been performed and several selective inhibitors have been identified (Bencharit et al., 2003a; Dominguez et al., 2014; Gilham et al., 2003; Shimizu et al., 2014). On the other hand, because of the demonstrated role of carboxylesterases in (pro)drug metabolism, the risk of undesirable drug-drug interaction should also be considered. For example, CES1 activates several angiotensin-converting enzyme (ACE) inhibitors (Thomsen et al., 2014), which are commonly used antihypertensive agents.

Some carboxylesterases, such as CES2, Ces2c and Ces1g, exhibit beneficial effects on lipid and carbohydrate metabolism when their activities are increased. Ces1g is a direct target of FXR (Xu et al., 2014a), and FXR activation is known to improve insulin sensitivity (Zhang et al., 2006), and has protective effects against hyperlipidemia (Bilz et al., 2006) and NAFLD (Carr and Reid, 2015). From this point of view, it is important to determine the human ortholog of Ces1g.

## CONCLUSION

Recent studies have demonstrated relevance of carboxylesterase activity to human metabolic disorders. The role of carboxylesterases as lipases and their functions in metabolism have attracted significant research interest. Importantly, several carboxylesterases possess lipase activity and appear to affect lipid metabolism and homeostasis in distinct or even opposing ways, such as human CES1 and CES2, or mouse Ces1d and Ces1g/Ces2c. This divergence of metabolic function could result from distinct substrate preferences of the different carboxylesterases. Given that the mouse expresses three-times the number of carboxylesterases compared to human it will be important to determine which mouse carboxylesterases are true functional orthologs of human carboxylesterases. When interpreting and translating research findings in pre-clinical carboxylesterase studies from mice to humans, differences of carboxylesterases between mice and human must be considered. The progress made so far suggests that several carboxylesterases are potential targets for the treatment of a number of human metabolic disorders. However, more studies are needed to thoroughly characterize the mechanisms by which carboxylesterases regulate lipid and energy homoeostasis.
